# Network Evolution of a Large Online MSM Dating Community: 2005–2018

**DOI:** 10.3390/ijerph16224322

**Published:** 2019-11-06

**Authors:** Chuchu Liu, Xin Lu

**Affiliations:** 1 College of Systems Engineering, National University of Defense Technology, Changsha 410073, China; 2 School of Software Engineering, Shenzhen Institute of Information Technology, Shenzhen 518172, China; 3 School of Business, Central South University, Changsha 410083, China

**Keywords:** social network, evolution, MSM, dating community, evolution pattern

## Abstract

Due to multiple sexual partners and low rates of condom use, the HIV infection rate among MSM (men who have sex with men) is much higher than that of the general population. In order to analyze the characteristics of online activities of MSM, and to understand the evolution of their social networks, in this study we collect a comprehensive dataset, covering the period from January 2005 to June 2018, from the largest Chinese online community, Baidu Tieba. We build an online dating network for MSM-related individuals in the gay-bar community, and analyze the network from static and dynamic aspects. It is found that there is a strong homophily regarding the cities where users reside when developing interactions with others, and that most network measurements tend to be stable at the later stages of evolution, while the size of the largest community fluctuates. This is an indication that the network is formed of rapidly flexible interactions which changes quickly. In comparison with studies on heterosexual networks, we find that the MSM dating network shows differences in many aspects, such as the positive degree-degree correlation and high clustering coefficient, suggesting different thinking and measures should be taken in the policy making of public health management towards the MSM population.

## 1. Introduction

By 2019, 32 countries and regions around the world had legalized same-sex marriage [1,2]. However, same-sex marriage is still not legally recognized in many countries, including China. Influenced by longstanding traditions such as ‘carrying on the family name’, homosexuality is still a sensitive issue in many countries or regions. In the Asia Pacific region, the sexual minority population is often one of the most marginal and vulnerable groups in society [3,4]. The MSM population refers to men who have sex with men [5,6,7], gay men, and some male bisexuals who have exhibit homosexual behavior, who currently account for approximately 5% of the total population in China [8,9]. Because MSM have multiple sexual partners and a low rate of condom use, the HIV epidemic and incidence among MSM are much higher than those among the general population [10]. HIV epidemics among MSM have continued to rise at an alarming rate in recent years [11,12]. Although recent statistics are not available, it was reported that the proportion of MSM with yearly reported new HIV/AIDS cases increased by more than 10 times, that is, from 2.50% in 2006 up to 28.25% in 2015 [13,14]. If the incidence of HIV cases is not properly controlled, the MSM population could easily become a medium of HIV transmission, prompting the spread of the virus more widely among the general population. Therefore, analyzing the characteristics of the dating activities of MSM, and understanding the social relationships in them, plays a crucially guiding role to establish and improve the health management mechanism for the MSM population.

Because of concerns about sensitivity and privacy, research data on hidden populations is generally difficult to access [15,16]. Moreover, the authenticity and accuracy of reporting data also challenge the validity and reliability of studies [17,18]. With the development of the internet and big data technology, people’s lives are increasingly inseparable from the internet. Individuals are able to carry out various social activities in internet communities, generating a huge amount of activity data and providing a new breakthrough for studies with traditional challenging issues. Massive amounts of internet open resource data have been increasingly used in the analysis and research of hidden populations [19,20,21,22]. Due to the existence of social discrimination, MSM lack reliable channels for making friends in the real world, and they are more dependent on social networking tools to find suitable friends or partners [23,24,25]. The anonymity of social networks has brought a good sense of security to this hidden population. Statistics in many studies have shown that the MSM population is very active online, far more than heterosexuals in seeking relationships [26,27,28]. Therefore, analyzing the dating characteristics and romantic relationships of MSM on the internet is an important perspective for understanding the MSM population.

In recent years, many studies have attempted to combine complex network technology with research in the online community of the internet to solve new problems [29]. For example, by using the community detection method of complex networks, Bruch and Newman studied the structure of heterosexual markets in the United States [30]. Holme et al. used the time series network analysis method to find out the structure and time evolution of a Swedish internet community, which is primarily intended for romantic communication [31]. Liu and Lu analyzed a popular HIV-themed online community in China, and found that the average topic similarity among members in HIV communities is positively correlated to network efficiency [32]. On the internet, MSM connect with each other every day in related communities, causing new interactions to be built, and there are new users joining. As a result, analyzing the online dating characteristics of MSM can be transformed into research on the user interaction network. However, there are few studies on the online interacting network of MSM, most of which analyzed only static characteristics [33,34,35]. Research on the long-term evolutionary characteristics of MSM dating networks is rare.

**Figure 1:**
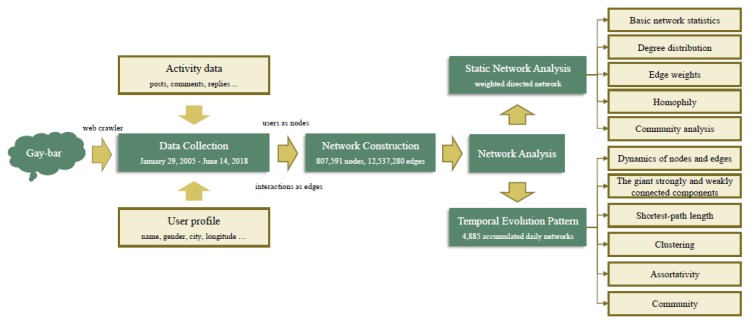
Overview of the research framework.

To fill in this gap of knowledge, we collected a comprehensive dataset in this study, covering the period from January 2005 to June 2018, from the largest sub-community related to MSM topics, gay-bar, in the world’s largest Chinese community (i.e., Baidu Tieba). We built the online interacting network for users on gay-bar, to analyze the romantic relationships of a Chinese MSM population on the internet. We carried out the analysis from static and dynamic aspects (Figure 1). We analyzed the static features of an online dating network for MSM—including nodal degree, edge weight, network communities, homophily, etc.—to investigate the social characteristics of the MSM population in the internet community. We also analyzed the evolution pattern [36,37] of the MSM online dating network, to develop an in-depth understanding of the changes in the online interaction networks of the MSM population in China over a 13-year period. In this study, we find out the interaction features as well as the evolution characteristics of the online MSM group, which is helpful to understand their social behavior, and essential for establishing and improving the health management mechanism on the MSM population.

## 2. Data and Methods 

With more than 1 billion registered users and over 300 million monthly active users, Baidu Tieba [38] is the largest online Chinese community in the world. It is a user communication platform based on users’ interest in millions of different topics, gathering massive numbers of users with common interests. These users who share the same hobby or focus on the same topic join subcommunities (called ‘bars’) for discussion and networking. To date, there are 22,736,986 subcommunities for different topics on Baidu Tieba, covering entertainment, games, novels, regions, life, and other various aspects. Because the bars attract a large number of accurate and segmented user groups, many popular bars have a great research value for analysis of a specific population, especially hidden populations with strong concealment, such as MSM, people with AIDs, sex workers, etc.

Gay-bar is the largest sub-community related to MSM on Baidu Tieba, on which millions of MSM-related users post their self-portraits, daily lives, viewpoints, etc., and communicate with each other. As of June 14, 2018, gay-bar had 4.67 million followers, and the cumulative number of posts had reached 300 million. The massive user data generated in this open community is of great significance for analyzing the characteristics of the MSM population in China. In this study, we developed a web crawler to collect from the latest to the oldest possible posts, as well as all following comments and replies under these posts, for a total of 13,012,892 records. All the data was distributed from January 29, 2005, to June 14, 2018.

Users’ activity data consists of the main posts, following comments, and replies. Posting on gay-bar refers to the user creating a new post with a specific title and specific content on a topic, called the main post. After the main post is created, its title and brief content are posted on the gay-bar homepage and are available for everyone to see. The gay-bar homepage refreshes and tops the latest posts. Then users can click on the links in the main posts to enter the detail pages, on which users can follow the post by commenting or replying to others. User data is related to users who have been active on gay-bar, mainly including the personal information that users fill in to register, as well as users’ activity information collected by Baidu Tieba, including the user’s name, gender, city, longitude, latitude, favorite community, etc.

There are mainly three types of social behaviors on gay-bar: posting, commenting, and replying. With the users as nodes, and the interactions between users as edges, we can construct the social network of this platform. Because the main purpose for users of gay-bar is making friends and seeking partners, this social network is actually a dating network. Since the first user joined gay-bar in 2005, new users and interactions have been added to the network accumulatively. By the end of the data collection period, the network contained 807,591 nodes and 12,537,280 edges. Each edge represents a unique contact between two users, with a unique ID and a timestamp indicating the day the contact was generated [39]. All edges are directed. With such data, we can focus on the evolution of the dating network on a daily basis and record the changes. Based on the initial network on the first day (January 29, 2005), new nodes and new edges of each day can be added to the initial network, in turn forming the next day’s dating network. Finally, we construct a total of 4,885 accumulated daily networks.

### 2.1. Ethics Approval and Consent to Participate

The study and Liu (2018) [32] were both supported by the Natural Science Foundation of China (91546203, 71771213) and approved by the Medical Ethical Committee of the Institutional Review Board (IRB) at Peking University (IRB00001052–16016). The study does not involve any physical, social or legal risks to the participants, and the data is anonymous, confidentiality of the participants’ information is strictly protected.

### 2.2. Data Availability

All data analyzed in this study are publicly available, all posts in the datasets can be collected on the website of Baidu Tieba (https://tieba.baidu.com/f?kw=gay). Other data that support the findings in this study are available from the corresponding author on reasonable request.

## 3. Results

### 3.1. Static Network Analysis

#### 3.1.1. Basic Network Statistics

We build the MSM dating network on gay-bar with users as nodes, and interactions between users as edges. In addition, we can use the number of interactions as the weight of the edges between each pair of users. A total of 5,132,933 weighted edges were found in the final dating network (network in the last day of the data collection), that is, 5,132,933 unique node pairs in which one node has interacted with another. In other words, the static analyses are all based on this directed weighted network. With calculations, we find the diameter of this dating network is equal to 12; that is, any two nodes on gay-bar need to go through 12 nodes at most to connect with each other. Moreover, the average shortest-path length is calculated as well, and we find the length of the gay-bar network is 4.23, which is consistent with the result that the average shortest-path lengths of many real-world networks are small [40,41]. Summaries of network statistics are presented in Table 1.

#### 3.1.2. Degree Distribution and Number of Posts

**Figure 2:**
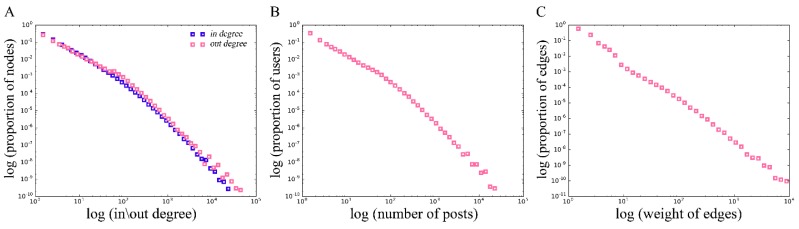
(**A**) The degree distribution of the MSM dating network. The horizontal axis represents the in-degree or out-degree, and the vertical axis represents the proportion of nodes with corresponding in-degree or out-degree in the network. (**B**) The distribution of the number of users’ posts on gay-bar. The horizontal axis represents the number of posts, and the vertical axis represents the proportion of corresponding users. (**C**) The distribution of the weights on the edges in the MSM dating network. The horizontal axis represents the edge weight, and the vertical axis represents the proportion of edges with the weight. All figures are in double-logarithmic (e) scales using log-bin [42,43].

The accumulated network has an average degree of 6.36; that is, on average, users in the gay-bar community interact with 6.36 others during the 13-year period. The degree distribution shows high heterogeneity, as has been found in many other large human social networks. It follows a truncated power law distribution (Figure 2A). It can be found that there are only 3 nodes with a degree greater than 10,000 in the dating network, and only 16,863 nodes with a degree greater than 100, accounting for approximately 2.1% of all nodes in this network. That is, there are only a very few nodes with relatively large degrees, and most nodes’ degrees are small, which means that interactions with other users are not frequent.

This finding corresponds to the posting characteristics of gay-bar users. The distribution of the number of user posts is shown in Figure 2B. As can be seen, because there are some ‘celebrity’ nodes on gay-bar, a few users post many times; e.g., user 1144404839 made 15,875 postings. Thus, this user’s chance of interacting with other users is greatly increased, causing them to be the nodes with large in-degrees in the network. In addition, some nodes are particularly positive in communications with other users, resulting in their large out-degrees in the network, such as user 910803194. All these ‘celebrity’ nodes and active nodes are responsible for the polarization of the nodes’ degrees. In contrast, most users do not post a lot (more than 53.8% of users posted fewer than 1,000 times, but the maximum number of user postings is larger than 1,000,000). We find that is because most users hang out on gay-bar only sometimes when they are free, and participate only in topics in which they are interested. This phenomenon also explains why the degrees of most nodes are very small in the dating network. Furthermore, we analyze the correlation between the degree and the number of posts, and find that the Spearman correlation coefficient (as s) between these two indexes is 0.714.

#### 3.1.3. Distribution of the Edge Weights

Figure 2C shows the distribution of the edge weights, which basically obeys a power law. The weight on the edges spans from 1 to 9,887, with the majority of edges containing only one interaction time (58.4%), and those with weights of less than 10 account for 98.1% of all edges. That is, most of the interactions between users are casual and infrequent, because most users interact occasionally with others only when they show great interest in the same topic at the same time, and have not established long-term stable friendships and frequent interactions. There is a positive correlation between the degree of the node and the sum of the weights on all his connected edges (the Pearson correlation coefficient, r = 0.939), indicating that users with more friends tend to be more active in interactions.

#### 3.1.4. Homophily

In early studies, it was discovered that most people are more inclined to make friends with those who are similar [44,45]. Moreover, in the recent exploration of network homophily, studies also illustrated that people prefer to make friends with people who have the same interests or hobbies [46,47,48]. It is believed that the same geographic location is an important factor for people’s social relationships, such as making friends or seeking partners [30,49].

We extract the user’s location information (either from the profile or the opt-to-public GPS location containing the latitude and longitude) as a new attribute of nodes in the network, named *loc*. We analyze whether the *loc* of the nodes has an effect on the formation of links between two different nodes. That is, we examine whether the nodes linked by the same edge are more likely to have the same *loc* rather than randomly. We use homophily to measure this probability, which is quantified as [50,51]

(1) $$H_{loc_{i}}=\frac{S_{loc_{i}\to loc_{i}}-P_{loc_{i}}}{1-P_{loc_{i}}},$$

where $H_{loc_{i}}$ is the homophily for nodes with property $loc_{i}$, $S_{loc_{i}\to loc_{i}}$ is the proportion of type $loc_{i}\to loc_{i}$ links among all links originating from type $loc_{i}$ nodes, and $P_{loc_{i}}$ is the proportion of type $loc_{i}$ nodes in the network. Consequently, $H_{loc_{i}}$ measures the exceeded likelihood of establishing interactions with users with the same location attribute ($loc_{i}$) for nodes of type $loc_{i}$. $H_{loc_{i}}=0$ means that all nodes interact with other nodes randomly and regardless of the $loc_{i}$ attribute. $H_{loc_{i}}$ =1 implies that all nodes with the $loc_{i}$ attribute are connected to friends with the same property. To measure the global homophily of the network, we use the average of all $H_{loc_{i}}$, represented as

(2) $$H=\frac{\sum_{i=1}^{k}H_{loc_{i}}}{k},$$

where *k* is the number of all unique *loc*.

With the definitions, we then measure the homophily of the MSM dating network in terms of the cities and provinces in which the users reside. The homophily for city is 0.356, indicating that, in addition to interacting with other users randomly, there is a 35.6% surplus of tendency for users to build interactions with people from their own city. The homophily for province is 0.478, greater than that of city, implying there is a 47.8% surplus of tendency for users to build interactions with people from their own province except random connections. These results suggest that although the internet has made people closer and brings a lot of convenience to daily communication, in the male homosexual dating network, similar geographic location is still an important condition considered by users for making friends and seeking partners. In addition, geographic homophily is more likely to promote the formation of interactions between users in the MSM community, which is consistent with the situation we observed in the content of user posts on gay-bar. We find that many posts are related to making friends and looking for a partner, where most users specifically mention their location, and put forward the strong desire to find male friends in the same region. For example, a popular post recently published on gay-bar states: “I’m 18 years old and in Harbin (capital city of Heilong Jiang Province), I’m here to look for a partner of type zero (the relatively feminine role in the MSM relationship).” Many users commented on his post, some indicating that their location is also Harbin. After the author interacted frequently with a commenter in the same location, both expressed their clear intention to become friends. We speculate that it is because the same geographic location is more conducive to meeting and dating internet pals for MSM, which is helpful for developing relationships on the internet that become romantic relationships in off-line real life, and more in line with users’ original intention and expectations for gay-bar. We also find in many posts many users made it clear that they did not want to maintain a virtual online relationship.

Furthermore, this discovery illustrates that users with the same geographic location are more likely to maintain close communication, thus forming a ‘friendship’, than maintain a long-term frequent interaction. However, only a few users can develop into this stable relationship, which explains why the number of edges with large weights is small. In most cases, in the MSM dating network, most users follow, comment, or reply to others only on certain topics (or posts) for the same interests; these social behaviors do not establish stable attention and interactions between users. Only a small number of users form in-depth relationships based on frequent interactions, and vice versa, close relationships create longer-term and frequent interactions.

#### 3.1.5. Community Analysis of the MSM Dating Network

**Figure 3:**
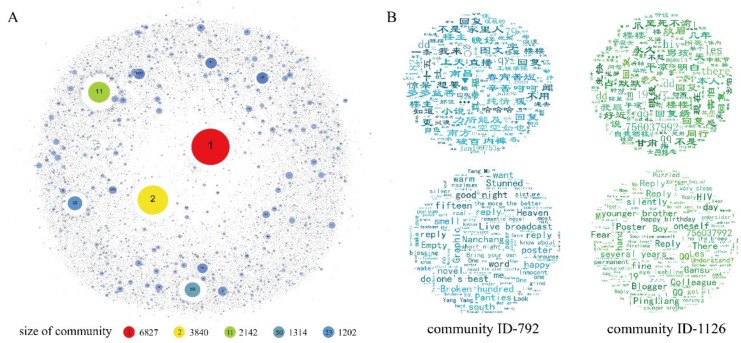
(**A**) The size of different communities in the gay-bar network. Each bubble represents a community, and the number in the bubble represents the community’s ID. The larger the bubble, the larger the corresponding community. (**B**) The word-cloud of topic keywords in community 792 and community 1126. The font size corresponds to the word frequency, and the English translation is presented below each of the word-cloud.

In this study, we use the Infomap algorithm [52,53], which is one of the most efficient, reliable, and accurate community detection methods [54,55], to detect the community structure in the MSM dating network. A total of 44,076 communities are found. The distribution of the communities’ sizes (the number of nodes in each community) is shown in Figure 3A. There are 812 communities (1.9%) with more than 100 nodes, and only 5 communities with more than 1,000 nodes. The number of nodes in 53.5% of the communities is less than 10. This result shows that the social groups in the MSM dating network are relatively small, and the relationships built by online interactions are relatively weak and non-stable. On gay-bar, users mainly pay attention to the posts they are interested in, and communicate only with others with the same purpose or characteristics, which cause most interactions in the network to be sparse and inconsistent.

To further understand the communities in the gay-bar network, text analysis is performed on the contents posted by users in each community. We use the latent Dirichlet allocation (LDA) model [56,57] to analyze the topics in the communities. We set each community to contain 10 main topics, each of which is represented by the top 20 keywords with maximum weights. Most keywords in these communities tend to be categorized into four classes: First, we find that the keywords in many communities include words referring to location (such as "Hunan", "Wuhan", "Chongqing", etc.), and number strings indicating users’ contact information (most are accounts on QQ or WeChat, and phone numbers). In addition, some common words that express personal information (such as “height”, “weight”, “photo”, “body”, etc.), as well as specific words that suggest users’ sexual orientation and roles, such as “top” and “bottom” are also frequent in the communities. According to the characteristics of the users’ postings on gay-bar, most of these words are used in the scene of MSM seeking friends and partners. Second, many keywords in the communities are related to games, stars, and sexual behaviors, mainly in daily chats among users. In addition, in many communities, numerous topic words are related to going to school, such as “university”, “student”, “school”, “high school”, etc. For example, “Hainan University” and “Donghua University” have high weights in community 792 (the ID of the community is 792), and “Xiamen University” in community 831 also shows a high weight, which indicates that a large portion of the MSM who hang out on gay-bar are students in school. This is consistent with the statistics published by recent studies that in China, 8.5% of male university students are MSM [58]. In this population, an increasing number of new HIV cases are reported: In 2016, 8 out of 10 HIV-infected Chinese university students were MSM [59]. Last, in the topic keywords of some communities, the frequency of “AIDS” and “HIV” is high as well, such as community 1126. This result suggests that HIV is still a common and anxious concern of MSM in China. Furthermore, it is also possible that some MSM on gay-bar have faced this condition or contracted the syndrome. The topic keywords in community 792 and community 1126 are shown in Figure 3B. As we can see, analysis of users’ contents in different communities helps us better understand the characteristics of the communities in the dating network, and discover the topics that are the daily focus and are discussed by this MSM online network.

The left graphic is keywords in community 792, and the keywords (translated into English) sorted by weight from large to small are warm, reply, poster, panties, wanna, live broadcast, Nanchang (city name), happy, naive in relationships, short night, family, hard, south, the more the better, do one’s best, college of engineering, video, Yang Mi (a popular actress), romantic, soul, white, university, the public, romantic novel, handsome guy, etc.

The right graphic is keywords in community 1216, and the keywords (translated into English) sorted by weight from large to small are hiv, reply, how long, poster, qq (a popular Chinese instant messaging software), Gansu (province name), silently, Pingliang (city name), same job, boy, lesbian, afraid, forever, draw eyebrows, eyeliner, close, self-sacrifice, bottom, college, Aids, hide, Lanzhou (city name), clavicle, bed, dentist, WeChat (a social application), make love, lymph, happy birthday, onlookers, Mid-Autumn Festival, etc.

### 3.2. Temporal Evolution Pattern of the MSM Dating Network

#### 3.2.1. Dynamics of Nodes and Edges

**Figure 4:**
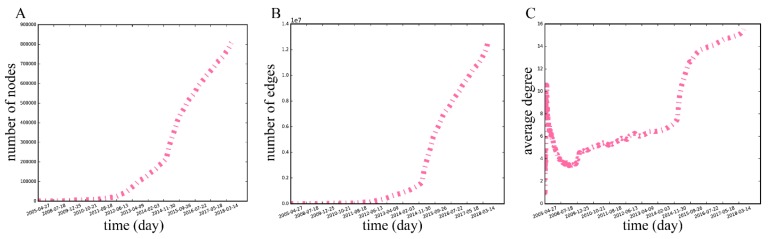
(**A**) The time evolution pattern of the number of nodes in the MSM dating network. (**B**) The time evolution pattern of the number of edges in the MSM dating network. (**C**) The time evolution pattern of the average degree in the MSM dating network. All horizontal axes represent the time in which the network was formed, and the vertical axes represent the value of indexes in the network for the corresponding time.

As time passes, the number of nodes and edges in the network increase, as shown in Figure 4. New users gradually joined the network at the beginning of stages: From April 27, 2005, to December 31, 2014, the number of users and edges reached 212,522 and 1,557,572, respectively. However, since 2015, the community has expanded rapidly with an average increase of 472 new nodes and 8707 edges each month. This upward trend is similar to the dynamics of the average degree (Figure 4C). However, we can see that there is a considerable decrease in the average degree after a sharp climb at the beginning of the network evolution, because increasing nodes make the density of the edges in the network smaller. The initial increase is perhaps because of the new users’ curiosity, making them engage in many social activities on gay-bar. Since then, the average degree of the network gradually increased, followed by a steep rise after 2015. Until the second half of 2016, the network average degree reaches around 14, and then continues to maintain a slow upward trend.

**Figure 5:**
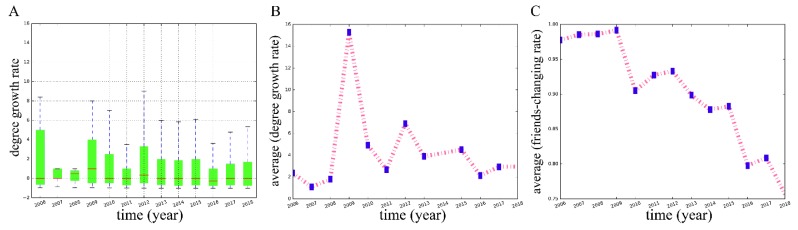
(**A**) The distribution of the degree growth rates of nodes each year. (**B**) The time evolution pattern of the average of the degree growth rates. (**C**) The time evolution pattern of the average of the changing friend rates. All these indexes are based on the annual separate networks, not the cumulative network (the MSM dating network).

To figure out the change pattern in the number of friends of nodes, the number of interactive friends per year (i.e., the degrees in the separate networks by year, not the cumulative network) for all users is calculated. We can see that the degree growth rates of most users are always greater than 0, shown in Figure 5A, which illustrates that the number of friends for most users keeps growing throughout the period. A positive correlation between the average of the degree growth rates (Figure 5B) and the node number growth rate in separate networks is discovered (*r* = 0.939), suggesting that the faster the number of nodes grows, the faster the number of friends for users grows. In addition, the annual change in user friends is analyzed. The proportion of different friends from the previous year for the same user is used to measure the rate that friends change. We find that the changing friend rates of most users are very high, approaching 1.0, indicating that for the majority, their communication objects tend to change by the next year. Most communications do not establish stable and continuous interacting relationships. However, this situation changed in the last few years. More and more users keep their friend lists with a smaller rate of change. As we can see from Figure 5C, the average changing friend rate generally maintains a downward trend, from 0.978 in 2006 to 0.757 in 2018. We find that there is a negative correlation between the average changing friend rate and the number of nodes (*s* = −0.901). The more nodes in the network, the smaller the probability that the users change the friends with which they interact.

#### 3.2.2. Dynamics of the Giant Strongly and Weakly Connected Components

**Figure 6:**
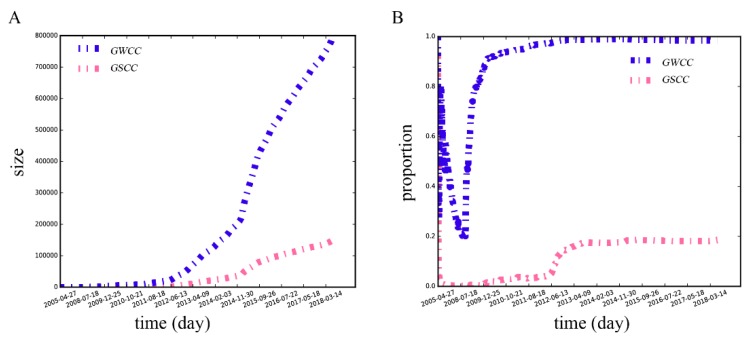
The size (**A**) and the proportion (**B**) of the GSCC and the GWCC in the MSM dating network. The horizontal axis represents the time when the network was generated. The vertical axis represents the size or the proportion of the GSCC and the GWCC, respectively.

Figure 6 shows the evolution of the giant strongly connected component (GSCC, in which there is a directed path from each node to all the other nodes) and the giant weakly connected component (GWCC, in which each node can either reach, or be reached by nodes from the GSCC) [60] in the MSM dating network, respectively. The GSCC and the GWCC grow slowly during the early stage, while the development trends of the proportions of the two components to the network show differences. The proportion of the GSCC quickly drops to around 0 and then maintains a gradual upward trend, while that of the GWCC climbs steeply to around 0.80, then falls to 0.2, followed by a rapid increase (consistent with the trend of the average degree at the early stage). After June 2012, the growth rates of both sizes of the GSCC and the GWCC begin to improve, the same as the proportion of the GSCC. Finally, the GSCC’s proportion stays at around 0.2, and the GWCC stays at around 1.0, while the sizes of the two components show faster growth, which is consistent with the increase trend of the number of the nodes and edges.

#### 3.2.3. Shortest-Path Length

**Figure 7:**
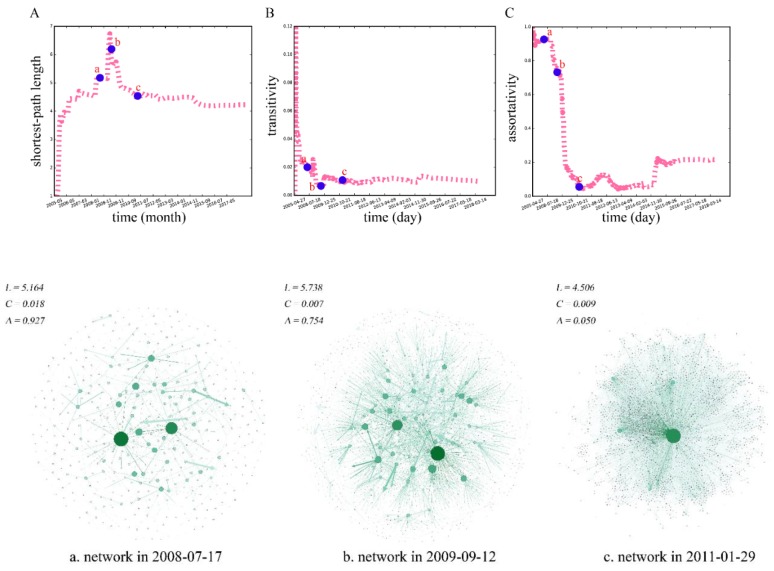
(**A**) The time evolution pattern of the average shortest-path length, L, in the MSM dating network. (**B**) The time evolution pattern of the clustering coefficient, C, in the MSM dating network. (**C**) The time evolution pattern of the assortativity, A, in the MSM dating network. a, b, and c are the networks corresponding to the marked points.

The average shortest-path length rises sharply before January 2006 (Figure 7A), and since then, it exhibits a ladder-type growth when the number of nodes and edges in the network is still relatively small. In April 2009, the length reaches the peak of the shortest-path length, that is, 6.73. The proportion of the giant weakly connected component is just in the fastest-growing period at this time. Thereafter, the average shortest-path length decreases gradually to about 4.20, and tends to be relatively steady, in which the number of nodes and edges in the network starts to soar. This result could suggest that there are denser interactions promoting users to be closer, although new nodes are still being added continuously. When the MSM dating network becomes more robust after 2015, the addition of new nodes and edges has less impact on the connectivity of the network, and the average shortest-path length remains at a small and stable value. This result is in line with the conclusion that the average shortest-path lengths of various human social networks are short.

#### 3.2.4. Clustering

The transitivity, or clustering coefficient [61], measures the probability that two neighbors of a vertex are connected, that is, the ratio of the triangles and connected triplets in the network,

(3) $$C=\frac{c\left(3\right)}{p\left(3\right)},$$

where *c(3)* represents the number of triangles actually present in the network, and *p(3)* represents the number of the largest possible triangles in the network.

For the MSM dating networks formed on different days, when the number of nodes and edges is small during the initial stage of the evolution, the clustering coefficient rapidly rises from 0 to the maximum value, 0.12. However, as the size of the network continues to increase, the clustering coefficient of the network begins to decrease, and finally reaches a stable value of 0.01. This indicates that even with the continuous increase in the nodes and edges, the ratio of triangles in the network remains basically stable to a certain value, and the clustering degree of the network does not change much during the later period. In other words, in the MSM dating network, the actual triangle accounts for 1% of all possible triangles. However, in a community populated largely by heterosexual individuals [31], the number of triangles would be much lower. Therefore, it can be claimed that the transitivity of a homosexual dating network, such as the dating network on gay-bar, is higher than that of a heterosexual network.

#### 3.2.5. Assortativity

For understanding the connection preference among MSM-related users in different periods, and further interpreting the tendency in seeking friends when Chinese MSM hang out online, we analyze the evolution pattern of the assortativity [62] of the MSM dating network. We calculate all the degree-degree correlations of the daily networks, and explore whether there is always considerable assortativity or disassortativity in different stages. In this study, the degree-degree correlation coefficient (R) proposed by Newman [63] is used to measure the network assortativity, quantified as:

(4) $$R=\frac{‹k_{to}k_{from}›-‹k_{to}›‹k_{from}›}{\sqrt{‹k_{to}{}^{2}›-‹k_{to}›{}^{2}}\;\sqrt{‹k_{from}{}^{2}›-‹k_{from}›{}^{2}}},$$

in which ‹.› denotes the average over arcs, $k_{to}$ is the degree of the vertex from which the edge leads, and $k_{from}$ is the degree of the vertex from which the edge starts. When R is greater than 0, it means that the MSM dating network is assortative, indicating nodes with large degrees in the network are more likely to generate connections with each other, and vice versa. That is, ‘celebrity’ or popular users on gay-bar tend to interact with users who are also famous or popular. Moreover, the absolute value of the coefficient represents the degree of the assortativity or disassortativity of the network: the more positive the coefficient, the more significant the assortativity; and the more negative the coefficient, the more significant the disassortativity.

The assortativity coefficients of the dating networks at different time are shown in Figure 7C. The coefficients are always greater than 0 with the network evolution. That is, the MSM dating network is always assortative, and nodes with large degrees tend to produce links with nodes that also have large degrees. At the beginning of the evolution, the network’s assortativity coefficient is equal to 1. It is because the number of users on gay-bar is small at that time, and the nodes are connected to each other, which lead to similar degrees among nodes. Then, with the continuous evolution, the number of nodes in the network continues to increase, and the assortativity coefficient gradually declines, and finally tends to a stable value, 0.22. In addition, the assortativity coefficients in different periods are always greater than 0; that is, the network is always assortative. The links in the network have a tendency to be created between nodes with a similar degree, which also suggests that the users on gay-bar are more inclined to communicate with users who are similar to them. Furthermore, MSM always prefer to seek similar friends or partners.

This assortativity of the MSM dating network is consistent with the classic scientific cooperation network and the actor cooperation network, but different from the online social networks for heterosexual romantic relationships [31]. Heterosexual social networks display a significant disassortative mixing for all types of degree-degree correlations [64,65]. This indicates that although all the networks are designed for romantic relationships on the internet, the online homosexual network and the heterosexual network show different characteristics: MSM have a tendency to look for friends and partners with similar degrees (popularity or personality) in an online dating network. However, in heterosexual relationships, there is disassortativity shown in the network, and nodes with larger degrees tend to establish connections with nodes that have fewer degrees; that is, popular users tend to look for heterosexual friends and partners with less popularity. Furthermore, it can be speculated that in homosexual romantic relationships, similarity is more important, but the difference or complementarity is more attractive in heterosexual relationships.

#### 3.2.6. Community

**Figure 8:**
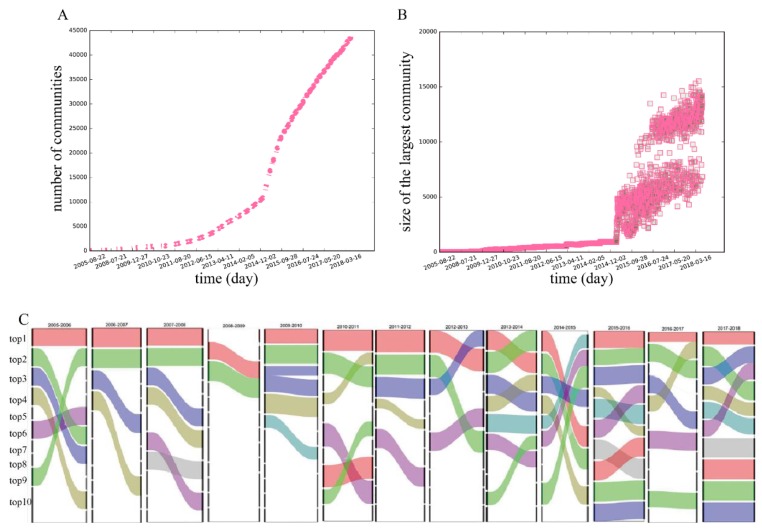
(**A**) The time evolution pattern of the number of communities in the MSM dating network. (**B**) The time evolution pattern of the size of the largest community in the MSM dating network. (**C**) The member flows in the 10 largest communities. The thickness of the stripe represents the proportion of the flow members; the thicker the line, the higher the flow ratio (between 0 and 1).

Analyzing the communities in the MSM dating network with time evolution is helpful to understand the social structure of MSM-related groups in different periods. For the 3696 networks formed on different days, the Infomap algorithm [52,53] is used to detect communities, and the number of communities in each network can be calculated. The number of communities in the daily networks is shown in Figure 8A. Over time, the number of communities maintains an upward trend, and the growth rate in 2015 is obviously improved, which is similar to the growth pattern in the number of nodes and edges.

We also analyze the size of the largest community in each daily network, as shown in Figure 8B. Surprisingly, it does not increase continuously, which is considerably different from other network indexes, such as the number of communities and the number of nodes. The size of the largest community fluctuates considerably after 2015, while the number of communities and nodes keeps climbing rapidly. This result suggests that during the later evolution period, the largest community continues to experience decomposition, integration, and reconstruction, which causes the size of the largest community to rise and fall. Through analyzing the relationship between the size of the largest community and the size of the network, we find that there is no direct correlation. More nodes in the network do not mean more or fewer nodes in the largest community. Furthermore, we try to explore the member flow in communities during the evolution period. Figure 8C describes the flow of nodes in the 10 largest communities. We can see that the node flows between different communities are frequent, and members in communities always change, indicating that the social circles of most users are not stable. This is also evidence of the flexible, rapidly changing, casual, and non-stable interactions in the MSM dating network.

## 4. Discussion and Conclusions

In this study, we collect the oldest to the newest possible activity data from the largest sub-community for MSM, gay-bar, in the largest Chinese community (i.e., Baidu Tieba), and construct an MSM dating network by figuring out the online interaction relationships between users. We also analyze the static characteristics of the MSM dating network, and the dynamic evolution pattern over the past 13 years, to explore the social features of the online MSM in China, as well as the changes.

We find that the nodal degree and the number of posts obey a power law-like heterogenous distribution, and that there is a positive correlation between them (*s* = 0.714). The weights on most edges are small, suggesting interactions between most users are casual and infrequent. The sum of the weights on connected edges also shows a positive correlation with the degree of the node (*r* = 0.939). To figure out whether the location is an important factor that affects online MSM making friends and seeking partners, the geographic homophily in the MSM dating network is calculated. We find that there is a strong homophily regarding the regions where the users reside when developing interactions with others (the homophily for province is 0.478). About communities in the network, it is found that most communities are small, indicating online MSM in China prefer small-scale social relationships. Most topic words in these communities can be categorized into four classes: personal information (including location and contact information), daily charts, school, and HIV (or AIDS).

To further explore the dynamic characteristics of MSM over time, we analyze the time evolution pattern of the MSM dating network, and construct the daily cumulative networks from January 29, 2005, to June 14, 2018. This network changes rapidly; the nodes and links are not stable over time. For most users, the number of interacting friends per year keeps growing. The change rate of their friends is very high, showing a negative correlation with the number of nodes in annual networks (*s* = −0.901). This reveals that the more nodes in the network, the more stable the friends of users. In addition, after a few changes during the previous period, the clustering coefficient levels off at around 0.01, which is higher than the transitivity of the online heterosexual social network. However, the assortativity of the MSM dating network also is quite different from that of the online heterosexual social network. The assortativity coefficient of the MSM dating network is always a large positive value, and remains at around 0.22 at the last period of evolution, while that of the latter is always less than 0, showing obvious disassortativity. Finally, we find that the number of communities increases over time, while the size of the largest community always fluctuates in the later period. Member flows between communities are frequent, which is an indication that the network is formed of rapidly flexible interactions that change quickly.

By analyzing the online dating network of the MSM-related population in China, this paper tries to figure out the static characteristics, as well as the dynamic evolution patterns, of the MSM dating network, to understand the social features of online MSM and the changes in the past 13 years. We found that the male homosexual social network shows differences with online heterosexual social networks in many aspects, such as the transitivity and assortativity. This result reveals the distinct evolving patterns of homosexual social networks in comparison to heterosexual social networks, and implies that decision makers need to adopt different perspectives and methods for development of public health policies targeting MSM. In addition, HIV is still a common and anxious concern of MSM in China. There should be more intervention strategies [66,67] in the HIV transmitted infections among the MSM population, such as identifying and training the most influential individuals (or opinion leaders) [68] within the MSM interacting network, or facilitating peer influence [69] to promote target health behaviors.

## Figures and Tables

**Table 1:** Static network measurements of the MSM dating network.

Number of Nodes	Number of Weighted Edges	Diameter	Average Shortest-Path Length	Average Degree	Edge Weights	Homophily for City (Province)	Number of Communities
807,591	5,132,933	12	4.23	6.36	(0,9887)	0.356 (0.478)	44,076
